# Antegrade Therapy for Management of Choledocholithiasis through Endoscopic Ultrasound-Guided Hepaticogastrostomy in a Patient with Surgically Altered Gastrointestinal Anatomy

**DOI:** 10.1155/2020/8866899

**Published:** 2020-11-12

**Authors:** Robert Dorrell, Katelyn Madigan, Swati Pawa, Rishi Pawa

**Affiliations:** ^1^Department of Medicine, Wake Forest School of Medicine, Winston-Salem, USA; ^2^Department of Medicine, Section on Gastroenterology, Wake Forest School of Medicine, Winston-Salem, USA

## Abstract

Endoscopic ultrasound-guided hepaticogastrostomy (EUS-HG) is a technique used to access the biliary tree in patients with surgically altered anatomy. Additionally, development of EUS-HG fistula permits intraductal therapy, thereby preventing patients from requiring surgery or percutaneous transhepatic biliary drainage (PTBD), thus decreasing morbidity. This clinical vignette describes an 83-year-old man with a history of gangrenous cholecystitis requiring cholecystectomy, partial gastrectomy, and Roux-en-Y gastrojejunostomy who presented to an outside hospital with abdominal pain and fever and found to have cholangitis and choledocholithiasis. He underwent two endoscopic retrograde cholangiopancreatography (ERCP) procedures at an outside hospital which were unsuccessful due to an inaccessible major papilla in the setting of the patient's surgically altered anatomy. On arrival to Wake Forest, the patient underwent EUS-HG with successful biliary drainage and resolution of cholangitis. He returned for ERCP three months later with balloon sphincteroplasty, cholangioscopy, and electrohydraulic lithotripsy (EHL) performed through the existing metal stent (hepaticogastrostomy), resulting in stone fragmentation and antegrade removal with balloon sweeps. Repeat cholangioscopy post-EHL and balloon sweeps showed complete duct clearance with no residual stones. The hepaticogastrostomy stent was subsequently removed, and the patient recovered without any complications.

## 1. Introduction

Endoscopic retrograde cholangiography (ERCP) is the cornerstone of accessing the biliary tree in patients requiring biliary drainage [1]. However, it is not feasible in all patients, such as those with surgically altered gastrointestinal anatomy (SAGA). Traditionally, patients with SAGA and acute biliary disease have either required invasive surgical techniques to reach the biliary tree or more often percutaneous transhepatic biliary drainage (PTBD), resulting in significant patient morbidity. Advances in the field of endoscopy have allowed for the development of novel techniques like endoscopic ultrasound-guided hepaticogastrostomy (EUS-HG), which provides patients with a safer and less-invasive option. EUS-HG involves the creation of a fistula between the stomach and the hepatic duct to access the biliary tree [2]. Through this gastrohepatic tract, ERCP can be performed for the diagnosis and management of biliary pathology [3]. This clinical vignette describes a patient with SAGA and subsequent successful treatment of choledocholithiasis and cholangitis via EUS-HG, cholangioscopy with electrohydraulic lithotripsy (EHL), and antegrade clearance of bile duct stones into the duodenum.

## 2. Case Description

An 83-year-old man with a history of gangrenous cholecystitis requiring cholecystectomy, partial gastrectomy, and Roux-en-Y gastrojejunostomy presented to an outside hospital with severe sepsis and recurrent unstable monomorphic ventricular tachycardia (requiring synchronized cardioversion) secondary to non-ST segment elevation myocardial infarction (NSTEMI). On examination, he was febrile (102.4°F) and hypotensive (systolic blood pressure of 85 mmHg), with generalized abdominal pain, nausea, and vomiting. Labs were notable for troponin, 1.31 ng/mL; total bilirubin, 4.0 mg/dL; aspartate aminotransferase (AST), 157 U/L; and alanine aminotransferase (ALT), 122 U/L. He was intubated and started on vasopressors. The following day, he was taken emergently to the cardiac catheterization lab during which a drug-eluting stent was placed for a coronary occlusion, and the patient was started on dual antiplatelet therapy. Following stabilization of the patient, a magnetic resonance cholangiopancreatography (MRCP) was obtained due to concerns for cholangitis, which demonstrated two large obstructing stones in the common bile duct (larger stone measuring 25 mm in diameter). He was formally diagnosed with severe acute cholangitis, grade 3, according to the Tokyo Guidelines [4]. He then underwent two unsuccessful ERCPs at an outside hospital (due to an inaccessible major papilla in the setting of SAGA) complicated by an additional cardiac arrest requiring life vest placement. On arrival to Wake Forest, he was noted to be febrile (102°F) and hemodynamically stable. After discussion with the patient and family regarding therapeutic options for choledocholithiasis and cholangitis including PTBD or EUS-HG, the patient elected to proceed with EUS-HG due to less morbidity and patient preference to avoid percutaneous drains. Given the patient's recent NSTEMI, cardiology was consulted and recommended holding dual antiplatelet therapy for no more than 3 days prior to the procedure to minimize the risk of stent thrombosis with immediate resolution 6 hours after procedure.

**Figure 1:**
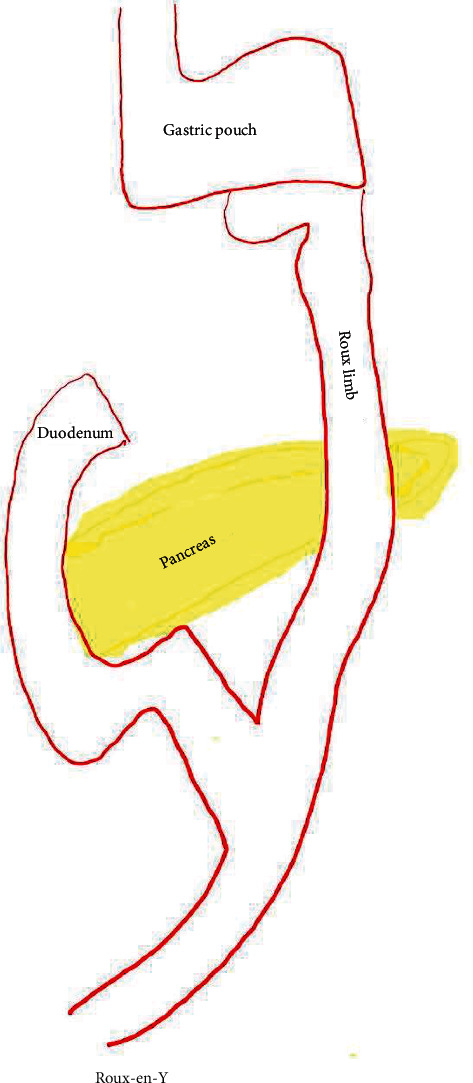
Schematic of surgically altered gastrointestinal anatomy: partial gastrectomy with Roux-en-Y gastrojejunostomy.

**Figure 2:**
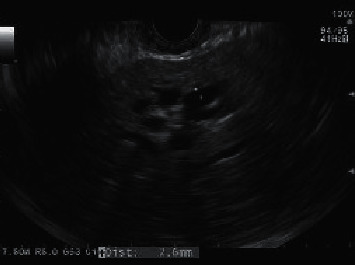
Endoscopic ultrasound showing a dilating segment 3 biliary radicle.

**Figure 3:**
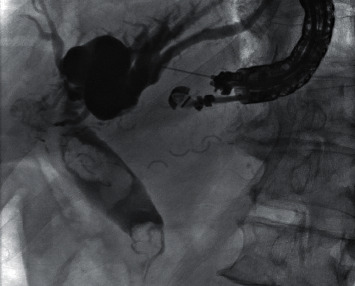
EUS-guided cholangiography showing a dilated biliary tree with choledocholithiasis.

**Figure 4:**
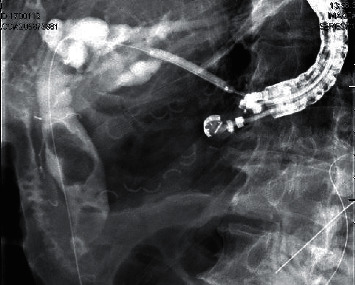
Balloon dilation of the HG tract.

**Figure 5:**
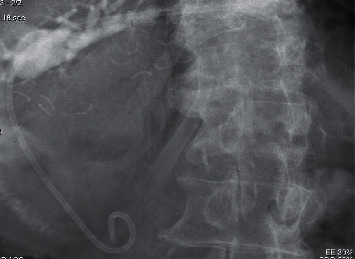
EUS-HG with placement of metal and double pigtail plastic stents.

Upper GI endoscopy revealed a gastric pouch with a patent Roux-en-Y gastrojejunostomy anastomosis (Figure 1). EUS was then performed using a linear echoendoscope (GF-UCT140-AL5; Olympus Medical Systems, Tokyo, Japan) connected to an ultrasound processor (Aloka Prosound Alpha 10; Hitachi, Tokyo, Japan). With the scope positioned in the gastric pouch, a dilated segment 3 branch was identified in the left lobe of the liver (Figure 2). The biliary radicle was punctured with a 19-gauge fine needle aspiration (FNA) needle (EchoTip Ultra HD, Cook Medical, Winston-Salem, NC). Following aspiration of the bile, contrast was injected through the needle for cholangiography. Cholangiogram showed diffuse intrahepatic ductal dilation and a dilated common bile duct measuring 18 mm in diameter with two large filling defects consistent with stones (Figure 3). A 0.025 inch in diameter and 450 cm in length straight tip VisiGlide 2 guidewire (Olympus, Tokyo, Japan) was then advanced through the needle into the biliary tree and past the major papilla into the duodenum. The hepaticogastrostomy tract was subsequently dilated using a 4 mm × 4 cm Hurricane biliary dilation balloon (Boston Scientific, Marlborough, MA, USA) (Figure 4). An 8 mm × 80 mm GORE VIABIL biliary stent (W. L. Gore Associates, Flagstaff, AZ) was placed in the left hepatic duct with the distal end in the gastric pouch. The metal stent was anchored by a plastic double pigtail stent (7 Fr × 18 cm) with the distal end of the plastic stent placed past the major papilla and the proximal end placed in the stomach (Figure 5). This procedure permitted decompression of the biliary tract while allowing the patient to recover from his acute infection.

**Figure 6:**
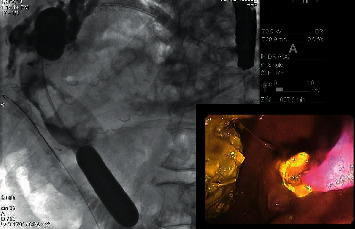
Antegrade balloon sphincteroplasty to facilitate stone removal.

**Figure 7:**
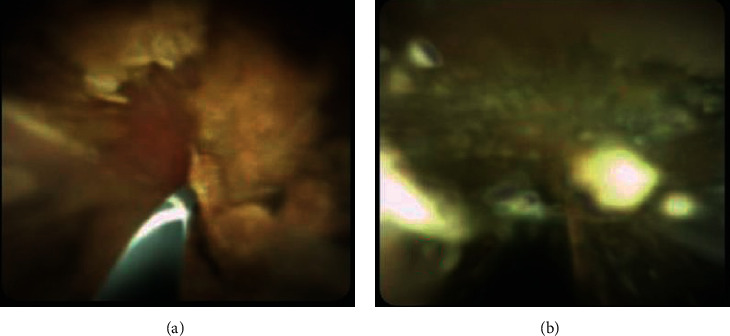
(a) Cholangioscopy showing CBD stones and (b) EHL resulting in common bile duct stone fragmentation.

**Figure 8:**
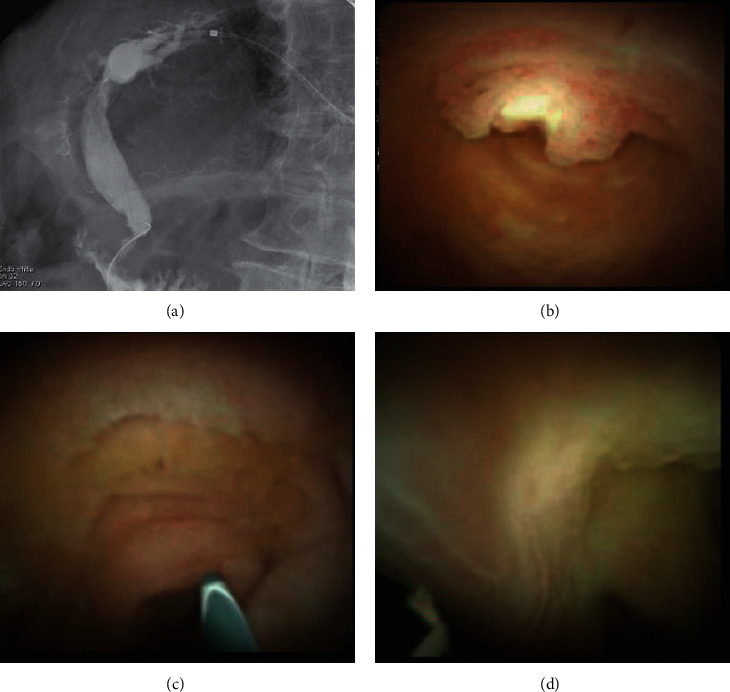
(a) Cholangiogram showing complete clearance of stones from the common bile duct. (b–d) Cholangioscopy showing normal distal common bile duct and ampulla with no residual stone disease.

The patient returned for ERCP 3 months after resolution of cholangitis and improvement of his cardiac function. Dual antiplatelet therapy was not held before procedure. Following removable of the plastic stent with a snare from the gastric pouch, the biliary tree was accessed via the left hepaticogastrostomy using a sphincterotome preloaded with a 0.025 inch × 450 cm VisiGlide 2 guidewire. Successful balloon sphincteroplasty was performed using a CRE (controlled radial expansion) wire-guided balloon (Boston Scientific, Marlborough, MA), and the diameter was gradually increased to 15 mm (Figure 6). Cholangioscopy was then performed using a SpyScope DS (Boston Scientific, Marlborough, MA) with visualization of two large stones in the common bile duct which were fragmented using EHL (Figure 7). The biliary tree was then swept with a 15 mm stone extraction balloon (Cook Medical, Winston-Salem, NC) with antegrade advancement of stone fragments past the ampulla into the duodenum with subsequent duct clearance (Figure 8). The patient was followed up in clinic 4 weeks later, and repeat LFTs were normal.

## 3. Discussion

This case presents a complex patient with significant comorbidities, multiple recent cardiac arrests, and prior partial gastrectomy and Roux-en-Y gastrojejunostomy who required urgent intervention for his cholangitis. However, traditional ERCP was unsuccessful due to his SAGA, and open surgery was not preferred as he was a poor surgical candidate given his underlying condition. PTBD was offered to the patient, but he refused due to preference against external drains. Though balloon enteroscopy has a high success rate and low adverse event rate, EUS-HG was preferred given the size of stones and the likely need for cholangioscopy and EHL [5, 6]. This successful intervention was accomplished by a two-step approach. The first procedure involved creation of hepaticogastrostomy with stent placement, allowing for biliary decompression and resolution of cholangitis. The subsequent procedure involved cholangioscopy with EHL, balloon sphincteroplasty, and antegrade clearance of stone fragments from the bile duct with balloon sweeps.

Numerous retrospective and prospective studies have compared EUS-HG with other interventions. One alternative to EUS-HG is percutaneous transhepatic biliary drainage (PTBD). Sportes et al. compared EUS-HG versus PTBD as a salvage procedure for failed ERCP and showed comparable clinical success (86% vs. 83%) [7]. Sharaiha et al. performed a systematic review and meta-analysis, which found that when compared to PTBD, endoscopic ultrasound-guided biliary drainage is associated with improved clinical success, lower rate of postprocedure adverse events, and fewer reinterventions [8]. Another alternative to EUS-HG is direct surgical exploration of the bile duct. This approach intrinsically carries a higher morbidity [9]. Clayton et al. performed a meta-analysis comparing surgical bile duct clearance versus endoscopic clearance and found no significant difference in outcomes [10]. Given the lack of improved outcomes with the more invasive techniques including PTBD and surgical exploration, EUS-HG for management of choledocholithiasis is the preferred alternative.

EUS-HG is a safe treatment approach for biliary drainage in patients with SAGA. To our knowledge, this is one of the few cases reported in the literature that describes successful performance of EUS-HG, antegrade cholangioscopy, and EHL for management of choledocholithiasis in SAGA [11–13]. Of the reported cases in the literature, the clinical success rate is greater than 90%. Due to its high clinical success rate and additional therapeutic options, EUS-HG should be included in the treatment algorithm for patients with surgically altered anatomy. This novel technique has shown positive short-term outcomes; however, randomized prospective studies are needed to formulate a consensus on the long-term efficacy and safety [3].

In conclusion, EUS-HG is a safe, effective, and less-invasive therapy that should be considered in the management of biliary pathology in patients with SAGA. In this patient with multiple surgical adaptations, numerous failed ERCPs, and significant comorbidities, EUS-HG was able to facilitate biliary drainage with successful treatment of choledocholithiasis and cholangitis.
